# Correction of lateral response artifacts from flatbed scanners for dual-channel radiochromic film dosimetry

**DOI:** 10.1093/jrr/rraa124

**Published:** 2021-01-22

**Authors:** Yuichi Akino, Hiroya Shiomi, Fumiaki Isohashi, Osamu Suzuki, Yuji Seo, Keisuke Tamari, Takero Hirata, Hirokazu Mizuno, Kazuhiko Ogawa

**Affiliations:** Oncology Center, Osaka University Hospital, Suita, Osaka 565-0871, Japan; Osaka University Graduate School of Meidcine, Suita, Osaka 565-0871, Japan; Osaka University Graduate School of Meidcine, Suita, Osaka 565-0871, Japan; Osaka Heavy Ion Therapy Center, Osaka 540-0008, Japan; Osaka University Graduate School of Meidcine, Suita, Osaka 565-0871, Japan; Osaka University Graduate School of Meidcine, Suita, Osaka 565-0871, Japan; Osaka University Graduate School of Meidcine, Suita, Osaka 565-0871, Japan; Osaka Rousai Hospital, Sakai, Osaka 591-8025, Japan; Osaka University Graduate School of Meidcine, Suita, Osaka 565-0871, Japan

**Keywords:** radiochromic film, lateral response artifact, dual color channel, quality assurance

## Abstract

In this study, we evaluated the inter-unit variability of the lateral response artifact for multiple flatbed scanners, focusing on the dual-channel method, and investigated the correction method of the lateral non-uniformity. Four scanners with A3+ paper-size and five scanners with A4 paper-size were evaluated. To generate the dose–response curves, small pieces of the Gafchromic EBT3 and EBT-XD films were irradiated, and five of the pieces were repeatedly scanned by moving them on the scanner to evaluate the lateral non-uniformity. To calculate the dose distribution accounting for the lateral non-uniformity, linear functions of the correction factor, representing the difference between the pixel values at offset position and the scanner midline, were calculated for red and blue color channels at each lateral position. Large variations of the lateral non-uniformity among the scanners were observed, even for the same model of scanner. For high dose, red color showed pixel value profiles similar to symmetric curves, whereas the profiles for low dose were asymmetric. The peak positions changed with dose. With correction of the lateral non-uniformity, the dose profiles of the pyramidal dose distribution measured at various scanner positions and that calculated with a treatment planning system showed almost identical profile shapes at all high-, middle- and low-dose levels. The dual-channel method used in this study showed almost identical dose profiles measured with all A3+ and A4 paper-size scanners at any positions when the corrections were applied for each color channel.

## INTRODUCTION

Radiochromic film dosimetry has been used for quality assurance (QA), including measurements of dose profiles and depth dose, evaluation of skin dose, and patient-specific QA of advanced radiotherapy techniques including intensity-modulated radiotherapy (IMRT), volumetric modulated arc therapy (VMAT) and stereotactic radiotherapy (SRT) [1–3]. Although recent 2D or 3D detector array devices have contributed time efficiency and volumetric evaluations, film dosimetry still has advantages in terms of high spatial resolution, especially for stereotactic treatments with steep dose gradient, and evaluation of the inter-leaf transmission and tongue-and-groove effects of a multi-leaf collimator (MLC) [4].

Currently, Gafchromic EBT3 (Ashland Inc, Wayne, NJ, USA) is widely used [5, 6]. The EBT3 film is used for the dose range 0.2–10 Gy. As of recently, type EBT-XD film is also available [7–10]. The EBT-XD film is designed for measurements of high dose, and the optimum dose range is from 0.4 to 40 Gy. For both EBT3 and EBT-XD films, the red color channel is reported to show the most sensitive response to the irradiated dose [8]. For pixel value (PV)-to-dose conversion, generally red, blue and green color channels are extracted from the scanned image and the red color channel is used (single-channel method). In contrast, the blue color channel is less sensitive to the irradiated dose, although both red and blue color data include the non-uniformity of the thickness of the sensitive layer. A dual-channel method, which uses the ratio of red and blue color data, was proposed by the film vendor to compensate for the non-uniformity of the film, although very few studies investigated this method [11]. Kairn *et al*. and Aland *et al*. reported that the blue-channel correction to the red-channel optical density increased the noise and uncertainties [12, 13]. A triple-channel method, which utilizes all red, green and blue colors for dosimetry, has shown excellent performance of accurate dosimetry [6, 14, 15].

Generally, the density of radiochromic film is evaluated as the transmission of light through films, and flatbed scanners are commonly used for image acquisition of the films. Many previous studies have reported that the PVs measured using flatbed scanners show variations associated with the position on the scanner [16, 17]. Some studies reported that the causes of these lateral response artifacts are an increase in the optical path length of the film at off-center position and the interaction of the polarization of light leaving the film and the mirror system guiding the light to the charge coupled device (CCD) sensor [18, 19]. This effect leads to incorrect measured data, and the impact is not negligible especially for patient-specific QA of large targets such as head-and-neck and pelvic regions. Many studies have investigated methods to compensate for the lateral response artifacts to improve the accuracy of film dosimetry [16, 20–22]. Although most of them were designed for a single-channel method, Lewis and Chan reported a correction method applicable for triple-channel corrections [23]. For multi-channel methods, each color channel must be carefully corrected. In addition, inter-unit variability of the lateral response artifact is also reported [23, 24]. Although flatbed scanners with A3+ and A4 paper-sizes have been widely used, the inter-unit variability of the A4 paper-size scanner has not been investigated.

We have used the dual-channel method combined with net optical density (OD_net_) calculations for routine QA. In this study, we evaluated the inter-unit variability of the lateral response artifacts for multiple flatbed scanners including both A3+ and A4 paper-sizes, focusing on the dual-channel method. We also investigated the correction method of the lateral response artifact for the dual-channel method.

## MATERIALS AND METHODS

### Handling of radiochromic films

For both Gafchromic EBT3 and EBT-XD films, the films were kept in the dark except during irradiations and scans. The size of the film was 20.3 × 25.4 cm^2^. The film was cut to obtain 13 pieces of film 4 × 4 cm^2^ for dose–response curves, and the remaining large sheet, 16.3 × 17.4 cm^2^, was used for verification (Supplementary Fig. 1, see online supplementary material). Before irradiations, the pieces of the films were scanned at the center of a flatbed scanner to evaluate the intra-sheet uniformity of the films, which was reported for Gafchromic EBT2 [25]. The films were scanned approximately 24 h after irradiation to reduce the influence of the variations of the intervals between the irradiation and image acquisition of the films.

To generate the dose–response curve, the small pieces were irradiated in solid water (Tough water, Kyoto Kagaku, Kyoto, Japan). Although the beam output was calibrated at 10 cm depth, the films were irradiated at 5 cm depth so that measurements could be repeated quickly. The source-to-surface distance (SSD) was 95 cm. In this study, a 6 MV photon beam with 10 × 10 cm^2^ field size was used. The monitor-units were determined by the tissue-phantom-ratio at 5 cm depth relative to that at 10 cm depth. The irradiated doses ranged from 0.25 to 10 Gy for EBT3 and from 1 to 40 Gy for EBT-XD films. One of the pieces was not irradiated to measure the base PV (0 Gy). The film pieces were scanned at the middle in the lateral direction of the scanner. The detail of the scanning procedure is described in the next section. On the scanned images, a region of interest (ROI) of 100 × 100 pixels was set for each film piece and the median PV in the ROI was measured for red and blue color channels.

### Flatbed scanners

Nine flatbed scanners were evaluated in this study. For A3+ paper-size (31.0 × 43.7 cm^2^), DS-G20000, ES-G11000 and ES-10000G (Seiko Epson Corp., Nagano, Japan) were tested. For A4 paper-size (21.6 × 29.7 cm^2^), GT-X970 and GT-X980 (Seiko Epson Corp.) were evaluated. The specifications and the number of units evaluated in this study are listed in Table 1. All models used the six-line (two lines for red, green, and blue color channels) CCD sensor. The latest models (GT-X980 and DS-G20000) used a white light-emitting-diode (LED) as the light source, whereas the older versions used a xenon lamp or cold cathode fluorescent lamp. The scanners were turned on at least 30 min before scanning images, and warm-up scans were repeated at least 10 times before scanning films. All functions of the scanner for correcting color and contrast were turned off. The resolution and the format of scanning images were 96 dpi and the 48-bit color TIFF format, respectively. For red, green and blue color channels, each pixel has 2^16^ levels. In this report, the parallel and perpendicular directions to the scanning of the CCD sensor were defined as the longitudinal (LONG) and lateral (LAT) directions, respectively. Films were placed with the longitudinal direction of the film parallel to the LONG direction. Two plates of non-reflecting glass were used to ensure that the films were flat and equidistant from the light source; the films were sandwiched by the plates and placed on the scanner. This technique was also used to correct the accurate dose–response curve from small film pieces. As shown in Fig. 1, the dose–response curve was smooth, indicating that the data would not be affected by the edge of the film pieces. Films for verification and scanner-correction data were scanned without limiting the ROI in the LAT direction to identify the positions on the scanner.

**Table 1:** Specification of the scanners^a^

Model	GT-X970	GT-X980	ES-10000G	ES-G11000	DS-G20000
Sensor	CCD	CCD	CCD	CCD	CCD
Light source	CCFL	W-LED	Xenon lamp	Xenon lamp	W-LED
Maximum size^b^	A4	A4	A3^+^	A3^+^	A3^+^
Number of units	2	3	2	1	1

^a^CCD = charge coupled device, CCFL = cold cathode fluorescent lamp, W-LED = white light-emitting-diode; ^b^A4, 21.6 × 29.7 cm^2^; A3+, 31.0 × 43.7 cm^2^.

**Table 2:** Accuracy of the approximation functions^a^. Note: mean ±standard deviation of the mean absolute errors of nine scanners are shown; the values were compared with the raw PVs

Film type	Color	(A) Lateral PV^a^ profiles	(B) Center–off^b^ axis correlation	(C) Lateral PV^c^ profiles
EBT3	Red	0.20% ± 0.05%	0.09% ± 0.03%	0.23% ± 0.19%
	Blue	0.25% ± 0.07%	0.04% ± 0.01%	0.25% ± 0.19%
XD	Red	0.23% ± 0.09%	0.09% ± 0.03%	0.25% ± 0.21%
	Blue	0.26% ± 0.08%	0.05% ± 0.03%	0.24% ± 0.17%

^a^(A) Quartic approximation function calculated from the horizontal position and the raw PVs; ^b^(B) linear approximation function calculated for PVs at center and off axis of the scanner; ^c^(C) quartic approximation function calculated from the horizontal position and PVs calculated using the function (B).

### Conversion from PV to dose

Analyses of PVs were performed using in-house software and Microsoft Excel. The OD_net_ is calculated as the logarithmic transformation of the ratio of pre-exposed (*I*_pre_) and post-exposed (*I*_post_) intensity values:

(1) \begin{eqnarray*} {\mathrm{OD}}_{\mathrm{net}}&=&{\mathrm{OD}}_{\mathrm{post}}-{\mathrm{OD}}_{\mathrm{pre}}={\log}_{10}\left(\frac{I_0}{I_{\mathrm{post}}}\right)-{\log}_{10}\left(\frac{I_0}{I_{\mathrm{pre}}}\right)\nonumber\\ &=&{\log}_{10}\left(\frac{I_{\mathrm{pre}}}{I_{\mathrm{post}}}\right) \end{eqnarray*}

where *I*_0_ represent the un-attenuated constant intensity from the light source [26]. Usually the PVs of un-irradiated film (PV_base_) are extracted from additional piece of un-irradiated film instead of measuring the values of the film before exposure. For a PV of an irradiated film (PV_IR_), the OD_net_ is calculated as follows:

(2) \begin{equation*} {\mathrm{OD}}_{\mathrm{net}}={\log}_{10}\left({\mathrm{PV}}_{\mathrm{base}}\right)-{\log}_{10}\left({\mathrm{PV}}_{\mathrm{IR}}\right) \end{equation*}

Previous studies [12, 13] have evaluated the OD_net_ for the dual-channel method (OD_net, RB_) using the following formula:

(3) \begin{equation*} {\mathrm{OD}}_{\mathrm{net},\mathrm{R}\mathrm{B}}=\frac{{\mathrm{OD}}_{\mathrm{net},\mathrm{R}}}{{\mathrm{OD}}_{\mathrm{net},\mathrm{B}}} \end{equation*}

where OD_net, R_ and OD_net,B_ represent the OD_net_ calculated for red and blue color channels, respectively. However, we also experienced that the OD_net,RB_ calculated with this formula did not provide appropriate results. Therefore, we previously developed the following formula calculating the OD_net,RB_ based on equation (2)2:

(4) \begin{equation*} {\mathrm{OD}}_{\mathrm{net},\mathrm{RB}}={\left[\frac{\log_{10}\left({\mathrm{PV}}_{\mathrm{R}}\right)}{\log_{10}\left({\mathrm{PV}}_{\mathrm{B}}\right)}\right]}_{\mathrm{base}}-{\left[\frac{\log_{10}\left({\mathrm{PV}}_{\mathrm{R}}\right)}{\log_{10}\left({\mathrm{PV}}_{\mathrm{B}}\right)}\right]}_{\mathrm{IR}} \end{equation*}

where PV_R_ and PV_B_ represent the PVs of the red and blue color channels, respectively. For each film piece, OD_net,RB_ was calculated using equation (4), and the irradiated dose was plotted against the OD_net,RB_ to generate the OD_net_-to-dose conversion curve [12, 26]. To convert the OD_net,RB_ value to dose, a quartic approximation function was calculated using a least-squares method. Because the conversion curve represents the net difference from the un-irradiated piece of film, the PV_base_ is also needed when converting PV to dose. Therefore, the dose–response calibration data included the OD_net_-to-dose conversion curve and PV_base_ for red and blue color channels.

### Curve fitting of lateral PV profiles

The method for correction of the lateral response artifact used in this study was similar to the method proposed by Lewis and Chang [23]. Five pieces of the film for generating calibration data were taped in line, and the image acquisitions were repeated by sliding the films in the LAT direction (Supplementary Fig. 2, see online supplementary material). The doses irradiated to the pieces (*D*) were 0-, 2-, 4-, 6- and 8-Gy for EBT3 films, whereas the doses were 0-, 6-, 10-, 20- and 40-Gy for EBT-XD films. On each scanned image, an ROI was set to the region of the films and the pixel data were copied onto a blank image to merge all scanned images. On the merged image, five ROIs were set to measure the profiles of the PVs of the same film pieces in the LAT direction. Quartic approximation functions of the lateral PV profile (PV_D (*x*)_) were generated using a least-squares method to interpolate the gaps. The origin of the profile was defined as the middle position of the scanner. For both red and blue color channels, a position *x* in LAT direction included five PVs for different doses (PV_*x* (*D*)_) which were calculated using the PV_*D* (*x*)_.

**Fig. 1.**
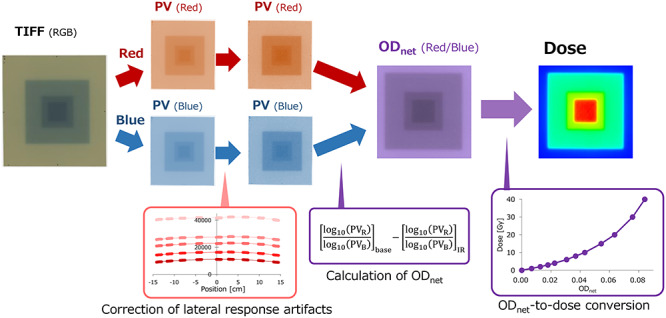
Scheme of the PV-to-dose conversion. The red and blue PVs are extracted from the scanned TIFF image and the scanner non-uniformity is corrected. The OD_net_ data are generated and the values converted to dose using the quartic approximation formula of the OD_net_-to-dose conversion curve.

### Correction of PVs for scanner non-uniformity

Supplementary Fig. 3, online supplementary material, illustrates the scheme for the correction of the lateral response artifact. The correction for a PV at position *x* (PV*_x_*) was performed with the following steps: (i) from five approximation functions generated in the previous section, one lateral PV profile (PV_D (*x*)_) providing slightly lower PV than PV*_x_* was selected, (ii) for the highest PV (0 Gy) and the selected lateral PV profile, the differences of the PVs from the values at the scanner midline (PV_Center_) were calculated, (iii) a linear function between the PV*_x_* and the difference from the PV_Center_ was calculated as the correction factor at position *x* (CF*_x_*), and (iv) the CF*_x_* calculated for PV*_x_* was added to the PV*_x_* to obtain the PV equivalent to the PV_Center_ (PV_Corr_). As shown in Supplementary Fig. 3C, the PV*_x_* and CF*_x_* demonstrated linear correlation. Therefore, the linear approximation function of CF was calculated:

(5) \begin{equation*} {\mathrm{CF}}_x=\alpha \bullet{\mathrm{PV}}_x+\beta \end{equation*}

(6) \begin{equation*} {\mathrm{PV}}_{\mathrm{Corr}}={\mathrm{PV}}_x+{\mathrm{CF}}_x \end{equation*}

where α and β represent the slope and intercept of the linear function, respectively. In clinical cases, however, the maximum doses vary with the prescribed dose. For cases with low prescribed dose, the linear function calculated for an entire range of doses may not be optimal. Therefore, the data points between the highest PV (0 Gy) and the PV slightly lower than PV*_x_*, representing a slightly higher dose, were selected for calculation of the linear function of CF*_x_*.

### Inter-unit variability of lateral non-uniformity among scanners

Using equations (5) and (6), the lateral PV profiles can be generated for an arbitrary PV_Center_. In this study, the film irradiations and image acquisitions were repeated four times on different days, and the images were acquired at three to four institutions for each measurement. Although the patterns of irradiations were the same for each day, the difference of elapsed time from the irradiation will affect the density of the five film pieces. For different scanners, the scanning conditions, including the intensity of the light source and the warm-up conditions, would also affect the scanned images. To investigate the inter-unit variations among the scanners correctly, the lateral PV profiles were generated for three arbitrary PV_Center_ values (low, middle and high doses), which were within the range of the PVs for all units. The PV_Center_ values of red color ranged from 14 000 to 38 000, and the values of blue color ranged from 17 000 to 25 000. The values were determined for type of film (EBT3 and EBT-XD), color channels (red and blue) and the size of scanners (A3+ and A4). To investigate the accuracy of the linear approximation function of equation (5), the lateral PV profiles were also generated for the PV_Center_ of the raw scanned images and the profiles were compared with the original data.

### Verification

To investigate the accuracy of the OD_net_-to-dose conversion with correction of the scanner non-uniformity, pyramidal dose distributions were evaluated using the Gafchromic EBT3 and EBT-XD films. Many previous studies have investigated a square field and clinical IMRT fields for such evaluations [16, 22, 23, 27]. Although the high-dose region can be evaluated with a square field, it is difficult to evaluate the accuracy of the middle- and low-dose at the penumbra regions. The dose distributions of the clinical IMRT plans depend on the commissioning of the treatment planning system (TPS), the complexity of the plan and the condition of the treatment unit. In this study, we have generated a pyramidal dose distribution using three square fields. A 6 MV photon beam generated by a TrueBeam linear accelerator (linac) (Varian Medical Systems, Palo Alto, CA, USA) was used. The jaw field sizes were 10 × 10 cm^2^, 5 × 5 cm^2^ and 3 × 3 cm^2^. For EBT3 film, the irradiated doses were 2 Gy for all three fields, whereas the doses for the EBT-XD film were 5-, 10- and 15-Gy for large, middle and small fields, respectively. The total doses at the central axis were 6 and 30 Gy for EBT3 and EBT-XD films, respectively. The films were scanned at various positions on the scanner in the LAT direction to investigate the impact of the scanner non-uniformity. The entire procedure of the film dosimetry is illustrated in Fig. 1. The dose distribution was also calculated on a virtual water-equivalent phantom using an Eclipse ver. 13.1 (Varian Medical Systems) TPS. The beam data of the analytical anisotropic algorithm (AAA) used for the calculation was modeled using the representative beam data of the TrueBeam linacs provided by the vendor.

### Dose distribution of VMAT plan

To investigate the feasibility of the proposed method for a more practical case, a VMAT plan of postoperative whole-pelvic radiotherapy for uterine cervical cancer was analyzed. The dose was calculated using the Eclipse TPS on solid water of 30 × 30 × 20 cm^3^, and the coronal dose distribution was extracted. The EBT3 film was irradiated with the same geometry to measure the coronal dose distribution. The irradiated film was scanned using a GT-X970 flatbed scanner, and the image was converted to dose using the dual-channel method with and without correction of the lateral response artifact. Gamma analysis was performed using in-house software with criteria of 3%/2 mm and threshold of 10% [28].

## RESULTS

To investigate the intra-sheet uniformity of the films, the film pieces were scanned at the scanner midline before irradiations. Measurements were performed on four different days. We evaluated the pieces prepared for 0–1 Gy irradiations to EBT 3 films and 0–4 Gy irradiations to EBT-XD films (Supplementary Fig. 1). For EBT3 films, the maximum differences were 0.70, 0.74 and 0.83% for red, green and blue color channels, respectively. For EBT-XD films, the maximum differences were 0.65, 0.63 and 0.72% for red, green and blue color channels, respectively. We also evaluated the intra-sheet uniformity in the LONG direction of the films. The maximum differences from mean values were 1.04% for all film types and color channels. These results indicate that the intra-sheet uniformity of the films was very low, and the variations of the PVs observed at different scanner positions will be primarily caused by the scanner.

Figure 2 shows the variations of the dual-channel method (OD_net, RB_) in the LAT direction for un-irradiated and irradiated films. The irradiated doses were 8- and 30-Gy for EBT3 and EBT-XD films, respectively. For both un-irradiated and irradiated films, some of the scanners showed variations in the LAT direction. The A3+-size scanners showed large variations especially at the off-center positions. Although the variations of the A4-size scanners were smaller than those of the A3+-size scanners, some scanners showed asymmetric variations.

**Fig. 2.**
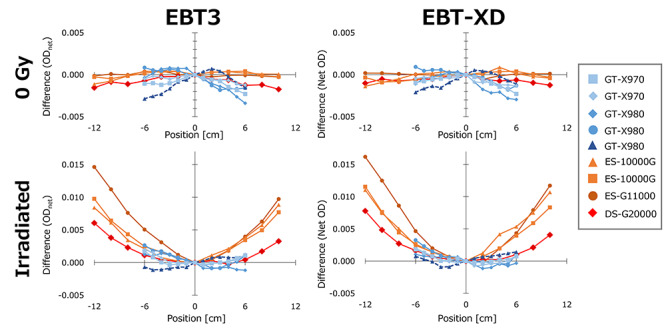
Lateral non-uniformity of the net optical density of dual-channel (OD_net,RB_) evaluated for nine scanners. Upper and lower rows show the data of the un-irradiated and irradiated films, respectively.

The accuracy of the approximation functions used in this study is summarized in Table 2. The mean differences of the approximated (A) lateral PV profiles from the raw profiles were within 0.26%, indicating that the quartic approximation was appropriate for the lateral PV profiles. The mean differences of the (B) center–off center correlation calculated using equation (4) were within 0.1%, indicating that the PVs at the center and off-center of scanners were linearly correlated. The mean differences between the (C) lateral PV profiles calculated with equation (6) and the raw profiles were within 0.25%. These data support the validity of the PV profiles calculated for an arbitrary PV_Center_.

Figure 3A and B show the variations of the PVs of EBT3 films calculated for an arbitrary PV_Center_ using equation (6). The variations from PV_Center_ were plotted. Large variations among the scanners were observed, even for the same model of scanner. Although the blue color channel showed modest difference between low and high doses, red color showed change of the PV profiles between low and high doses. For high dose, the red color showed profiles close to symmetric curves, whereas the profiles for low dose were asymmetric. Figure 3C shows the peak positions of the PV profiles. For low dose, some of the scanners showed the peak positions of red color profiles at off-center. The peak positions of these scanners moved with increasing dose. In contrast, most scanners showed a very small change in the peak positions of blue color, although a few scanners showed changes with dose. The EBT-XD films showed similar results (Fig. 4).

**Fig. 3.**
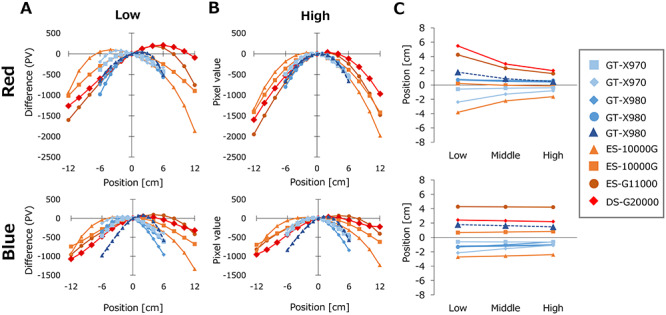
Lateral non-uniformity of the PVs of nine scanners evaluated for EBT3 films. The values were calculated for arbitrary PVs at the center of scanners (PV_Center_). (**A**) PV profiles calculated for low PV_Center_. (**B**) PV profiles calculated for high PV_Center_. For (A) and (B) the differences of the PVs from PV_Center_ were plotted. (**C**) The peak positions of the PV profile curves.

**Fig. 4.**
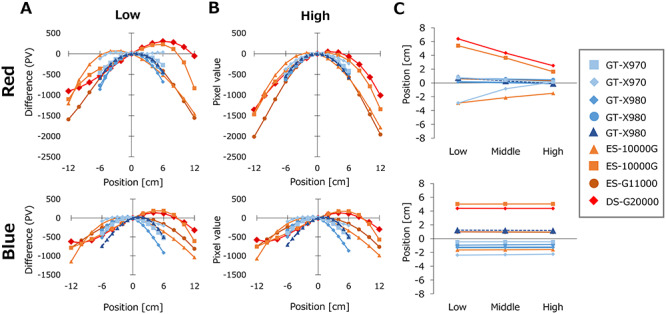
Lateral non-uniformity of the PVs) of nine scanners evaluated for EBT-XD films. The values were calculated for arbitrary PVs at the center of scanners (PV_Center_). (**A**) PV profiles calculated for low PV_Center_. (**B**) PV profiles calculated for high PV_Center_. For (A) and (B) the differences of the PVs from PV_Center_ were plotted. (**C**) The peak positions of the PV profile curves.

Figures 5 and 6 show the lateral dose profile of the pyramidal dose scanned with shifting the EBT3 and EBT-XD films, respectively. In Figs 5A and 6A, example dose profiles were plotted against the absolute coordinate on the scanner. The dose without corrections increased, especially at the off-center positions. In contrast, the corrected dose profiles showed similar profile shapes at any position. In Figs 5B and 6B, the horizontal axis represents the position relative to the central axis of the beam. Points colored red represent the dose profiles calculated with the Eclipse TPS. The un-corrected dose profiles showed large variations especially at middle- and high-dose levels, although the variation in the low dose level was modest. Figures 5C and 6C show the dose at the center of the pyramidal dose distribution plotted against the film positions on the scanner in the LAT direction. The dose profiles of a film placed on the position closest to the scanner midline was used as reference, and the dose differences from the reference were plotted. At the off-center positions, the doses of the un-corrected profiles increased up to 47%. In contrast, the corrected dose profiles showed very small variations, and the means ±standard deviation (SD) (range) were −0.6% ±1.2% (−3.0 to 2.5%) and −0.6% ±2.1% (−4.5 to 3.8%) for EBT3 and EBT-XD films, respectively.

**Fig. 5.**
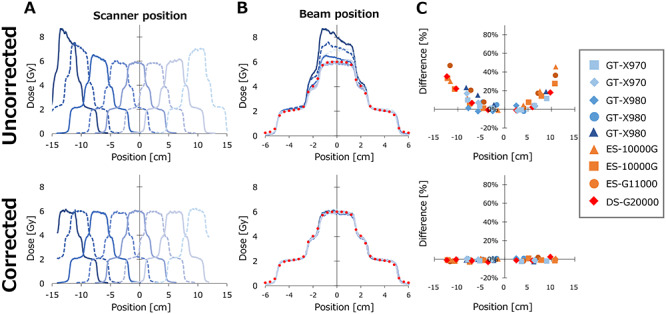
The pyramidal dose profiles of Gafchromic EBT3 film measured at various scanner positions. Upper and lower panels represent the profiles without and with correction of the lateral response artifacts, respectively. (**A**) Example of the profiles plotted with the positions on the scanner. (**B**) Profiles of all scanners plotted with the position relative to the central axis of the beam. Points represent the dose calculated with the Eclipse. (**C**) The center dose of the pyramidal dose distribution at the central axis of the beam was plotted with the positions on the scanner. The dose profiles of a film placed on the position closest to the scanner midline was used as reference, and the dose differences from the reference were plotted.

**Fig. 6.**
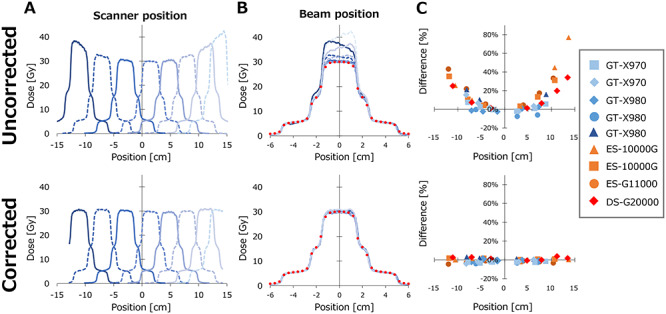
The pyramidal dose profiles of Gafchromic EBT-XD film measured at various scanner positions. Upper and lower panels represent the profiles without and with correction of the lateral response artifacts, respectively. (**A**) Example of the profiles plotted with the positions on the scanner. (**B**) Profiles of all scanners plotted with the position relative to the central axis of the beam. Points represent the dose calculated with the Eclipse. (**C**) The center dose of the pyramidal dose distribution at the central axis of the beam was plotted with the positions on the scanner. The dose profiles of a film placed on the position closest to the scanner midline was used as reference, and the dose differences from the reference were plotted.

We also evaluated the doses of the profiles at 2- and 4-cm beam positions, representing the doses at middle- and low-dose levels, respectively, by normalizing the profiles at the central axis of the beam. In the middle dose level, the maximum variations were −18.5 and −21.1% for EBT3 and EBT-XD films, respectively. In the low dose level, maximum variations were −29.2 and −28.4% for EBT and EBT-XD films, respectively. The corrected dose profiles showed small variations. In the middle dose level, the means ±SD (range) were −0.1% ±1.8% (−3.7 to 5.0%) and 0.0% ±2.3% (−3.7 to 5.8%) for EBT3 and EBT-XD films, respectively. In the low dose level, the means ±SD (range) were 0.9% ±2.2% (−5.0 to 5.3%) and 0.9% ±2.9% (−3.1 to 9.8%) for EBT3 and EBT-XD films, respectively.

Figure 7A shows the coronal dose distribution of a whole-pelvic VMAT plan. The dose distribution converted from EBT3 film without correction of the lateral non-uniformity (un-corr) showed lower dose in the left side. Figure 7B shows the lateral dose profiles extracted from the positions illustrated in Fig. 7A. Although the un-corrected film doses showed different profile shape from the TPS, the corrected film dose showed good agreement with the TPS. The gamma passing rates at 3%/2 mm were 59.1 and 97.1% for un-corrected and corrected films, respectively.

**Fig. 7.**
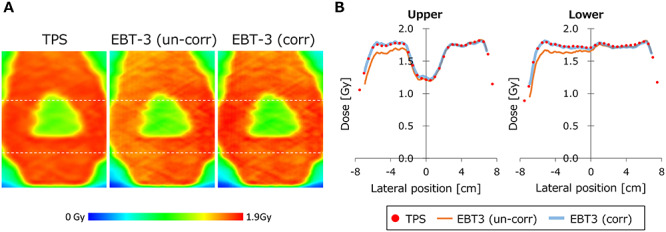
(**A**) Coronal dose distribution of a VMAT plan calculated with a TPS, measured with EBT-3 and converted without correction of lateral response artifact (un-corr) and with correction (corr). (**B**) Lateral dose profiles. The positions of the profiles are illustrated in (A) as dashed lines.

## DISCUSSION

In this study, we have investigated the inter-unit variability of the lateral response artifacts among nine flatbed scanners focusing on the dual-channel film dosimetry technique. Previously, some studies reported that the dual-channel methods increased the noise and uncertainties [12, 13]. They calculated the dual-channel OD_net_ using equation (3), but the values would be strongly affected by the uncertainties of the blue color channel especially at low doses. In this study, we calculated the dual-channel OD_net_ using a modified formula (equation 4) and showed good agreement between the TPS-calculated and film-measured doses derived from all scanner units at low-, middle- and high-dose levels. With the lateral response artifacts for the dual-channel method, almost identical dose profiles were obtained from the films scanned at any positions on the scanner. The VMAT test also showed good agreement between the TPS-calculated and film-derived dose distributions with correction of lateral scanner non-uniformity. We consider that the dual-channel method proposed in this study is useful for film dosimetry including commissioning and patient-specific QA of IMRT/VMAT treatments.

Previous studies also evaluated several flatbed scanners to investigate the variability among them. Lewis and Chang evaluated four flatbed scanners with A3+ paper-size for film dosimetry using EBT3 films and reported the variability of the correction coefficient among them [23]. They also evaluated three scanners and reported similar results for EBT-XD films [24]. Although scanners with A4 paper-size have also been used for film dosimetry, inter-unit variability among scanners with A4 paper-size has not been investigated, especially for multi-channel film dosimetry. This study showed that the A4 paper-size scanners also showed dose-dependent and asymmetric patterns of lateral response artifacts, and the effects were appropriately compensated for by the correction of dual color channels. The A4 paper-size scanners seemed to have smaller lateral scanner non-uniformity than that of A3+ paper-size scanners because of their smaller size in the LAT direction (Fig. 2). However, the VMAT QA test showed that the impact of the lateral non-uniformity of the A4 paper-size scanners was very large and not negligible (Fig. 7). Although the impacts were scanner-dependent, some scanners showed variations > 5% even at 3 cm from the scanner midline. The lateral scanner non-uniformity should be characterized before commissioning for clinical use. For small target cases such as SRT for brain metastases, high gamma passing rate is expected without corrections because the gamma indices of pixels at steep dose gradient will be reduced by the parameter of distance. When evaluating the beam profiles for machine QA, corrections are necessary for all collimator sizes because the peak positions of some scanners are not at the scanner midline.

As illustrated in Figs 3 and 4, red and blue color channels showed different patterns of the lateral response artifacts. For low dose, most scanners showed lateral non-uniformity in an asymmetrical manner. Interestingly, some of the scanners showed different profile shapes and peak positions between red and blue colors. In addition, the profile shapes of red color changed greatly with dose, whereas blue color profiles showed very small changes with dose. Large inter-unit variations in the profile shape change were also observed. This study included scanners with fluorescent lamp (GT-X970, ES-10000G and ES-G11000) and scanners with LED light source (GT-X980 and DS-G20000). However, the variations observed among scanner units did not depend on the models of the scanner. With correction of lateral non-uniformity, all scanners provided appropriate dose profiles at any scanner positions. Lárraga-Gutiérrez *et al*. previously compared an LED-type flatbed scanner (Epson V800) and a cold cathode fluorescent lamp-type scanner (Epson 11000XL) [29]. They reported that the overall performance of these two scanners were comparable, although differences in the color-dependent sensitivity and the lateral non-uniformity were observed.

In this study, we used the dual-channel method combined with the OD_net_. Because the dual-channel method uses both red and blue color channels, the different patterns of the profile shape changes between the two color channels would affect the lateral non-uniformity of the converted dose distributions. To correct the lateral non-uniformity properly, the PV profiles of the scanner should be collected with small spacing. As illustrated in Supplementary Fig. 2, we scanned the film pieces by sliding them with ~2–3 cm spacing, and the quartic fitting curves were calculated using a least-squares method. The mean absolute error between the fitting curves and the original lateral profiles were within 0.3%, indicating that the quartic approximation accurately reproduced the original data.

Many studies have investigated the correction methods for the lateral response artifact of flatbed scanners, and they evaluated the feasibility of the method by evaluating the dose profile of open field [16, 24]. However, the accuracy of the correction at middle- and low-dose levels cannot be evaluated from the penumbra region. We evaluated the pyramidal dose distribution and showed that the lateral response artifact was appropriately corrected at middle- and low-dose regions. Menegotti *et al*. [20] and Chang *et al*. [21] also evaluated the pyramidal or inverse pyramid dose patterns, although they did not move the films on the scanner. Lateral response artifact for triple channel corrections has also been reported. Micke *et al*. reported that the triple-channel technique mitigated the lateral response artifact [14]. Lewis and Chang reported the correction technique to be applicable for triple-channel corrections [23]. Our method also corrected the non-uniformity of both red and blue color channels before calculating the OD_net_ and provides adequate dose distributions with simpler calculations.

We repeated film measurements four times, and the images of the films were acquired at three to four institutions for each measurement. Therefore, the time interval between the irradiation and the image acquisitions were not exactly the same. In addition, repeated scanning would affect the density of the films, although the impact of scans on the relative dose distribution would be small. Paelinck *et al*. and Lárraga-Gutiérrez *et al*. reported that the impact of 50 consecutive scans on the PVs were within 2% [16, 29]. In this study, the PV_base_ of the dose–response curve was used for conversion of the film image of the pyramidal dose distribution to OD_net_. The films for the calibration data and that of pyramidal dose distribution were irradiated at the same time (within 30 min). This procedure is expected to reduce the uncertainties due to the variations in the time intervals.

We previously measured the dose–response of a single production lot of EBT3 film weekly for 13 weeks (Supplementary Fig. 4) and found that the median variations of the dual-channel dose–response curve from the initial measurement were within 5% (range −4.3 to 1.3%). The variations seemed random, probably because of the scan-to-scan variability of the scanner [30]. Dreindl *et al*. also investigated the change of the dose–response curve in the long-term and reported that the curve of Gafchromic EBT3 film showed a median variation of 5.0% over 16 months [31]. For evaluation of relative dosimetry, we consider that frequent updating of the dose–response curve is not needed. For absolute evaluations, generating calibration data for each measurement would improve the accuracy by reducing the uncertainties due to the scan-to-scan variations and the intervals between the irradiation and scans [27].

## CONCLUSIONS

In this study, we demonstrated that the lateral response artifacts showed inter-unit variabilities for A3+ and A4 paper-size flatbed scanners even for the same models. The shape and the peak position of the PV profile curve varied with dose, and the patterns of the dose-dependent change of the artifacts were not the same for red and blue color channels. These findings are in agreement with previous studies. The A4 paper-size scanners also showed significant inter-unit variabilities. Although the lateral response artifacts of the A4 paper-size scanners seems modest compared to the A3+ paper-size scanners, appropriate corrections are needed for accurate dosimetry. The dual-channel method used in this study showed almost identical dose profiles measured for all scanners at any positions on the scanners if the corrections were applied.

## Supplementary Material

Suppl_Figure_1_R1_rraa124

Suppl_Figure_2_R1_rraa124

Suppl_Figure_3_R1_rraa124

Suppl_Figure_4_R1_rraa124
