# Supplementary figures and images for: Correction of lateral response artifacts from flatbed scanners for dual-channel radiochromic film dosimetry
Source: J Radiat Res. 2021 Jan 22;62(2):319–28. doi: 10.1093/jrr/rraa124 (PMC7948896; doi:10.1093/jrr/rraa124)

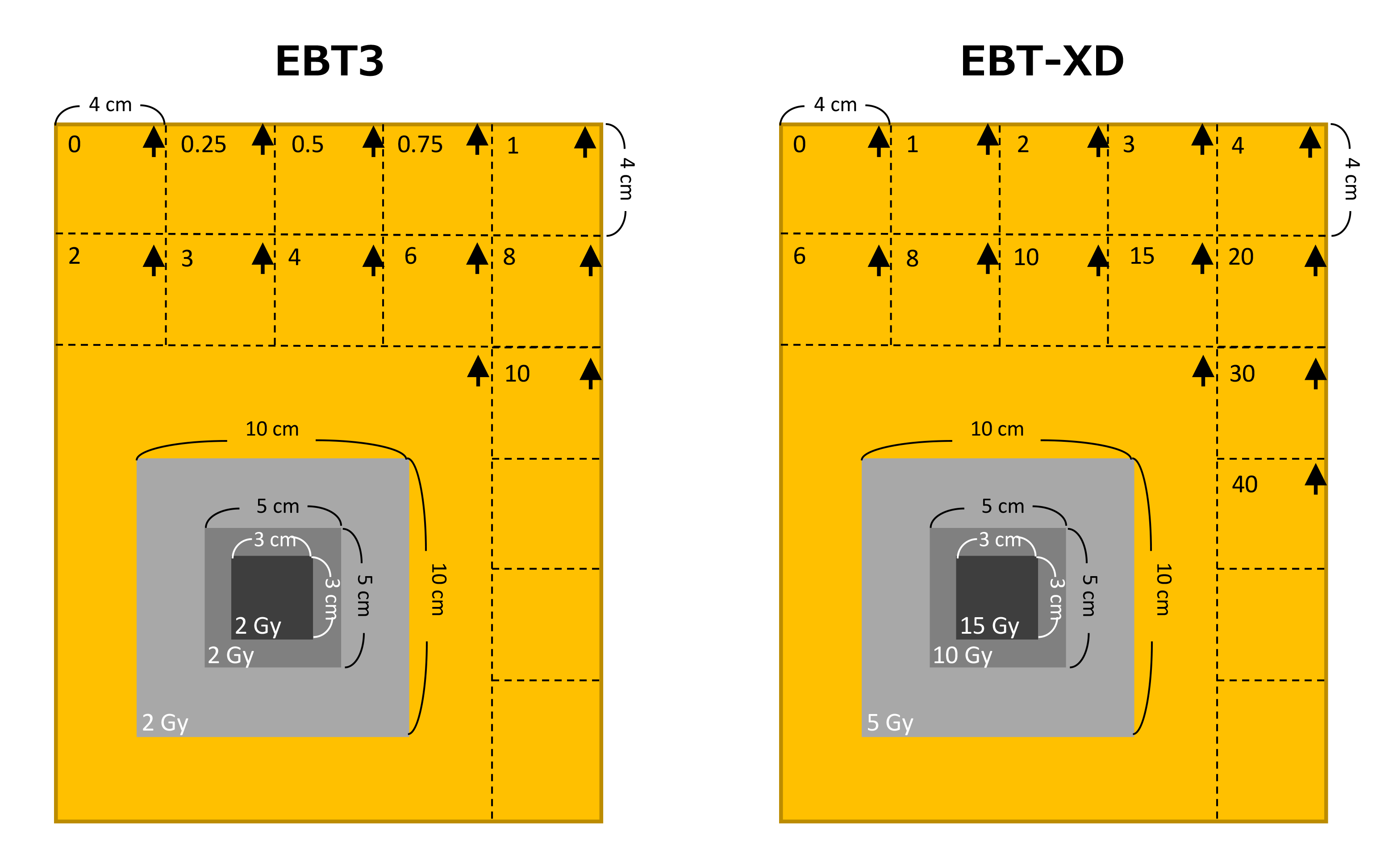

Supplement: Suppl_Figure_1_R1_rraa124 [file suppl_figure_1_r1_rraa124.png]

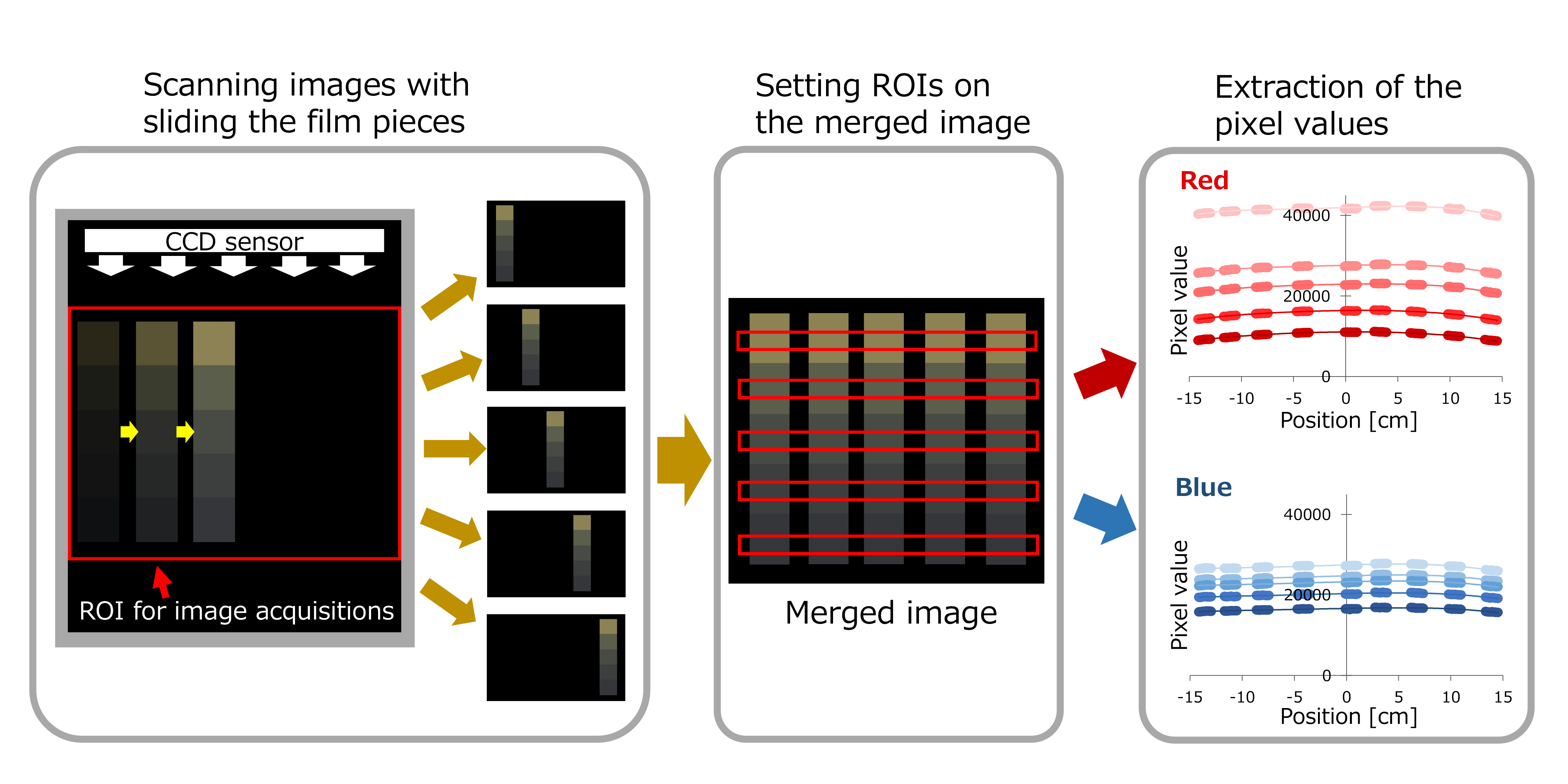

Supplement: Suppl_Figure_2_R1_rraa124 [file suppl_figure_2_r1_rraa124.png]

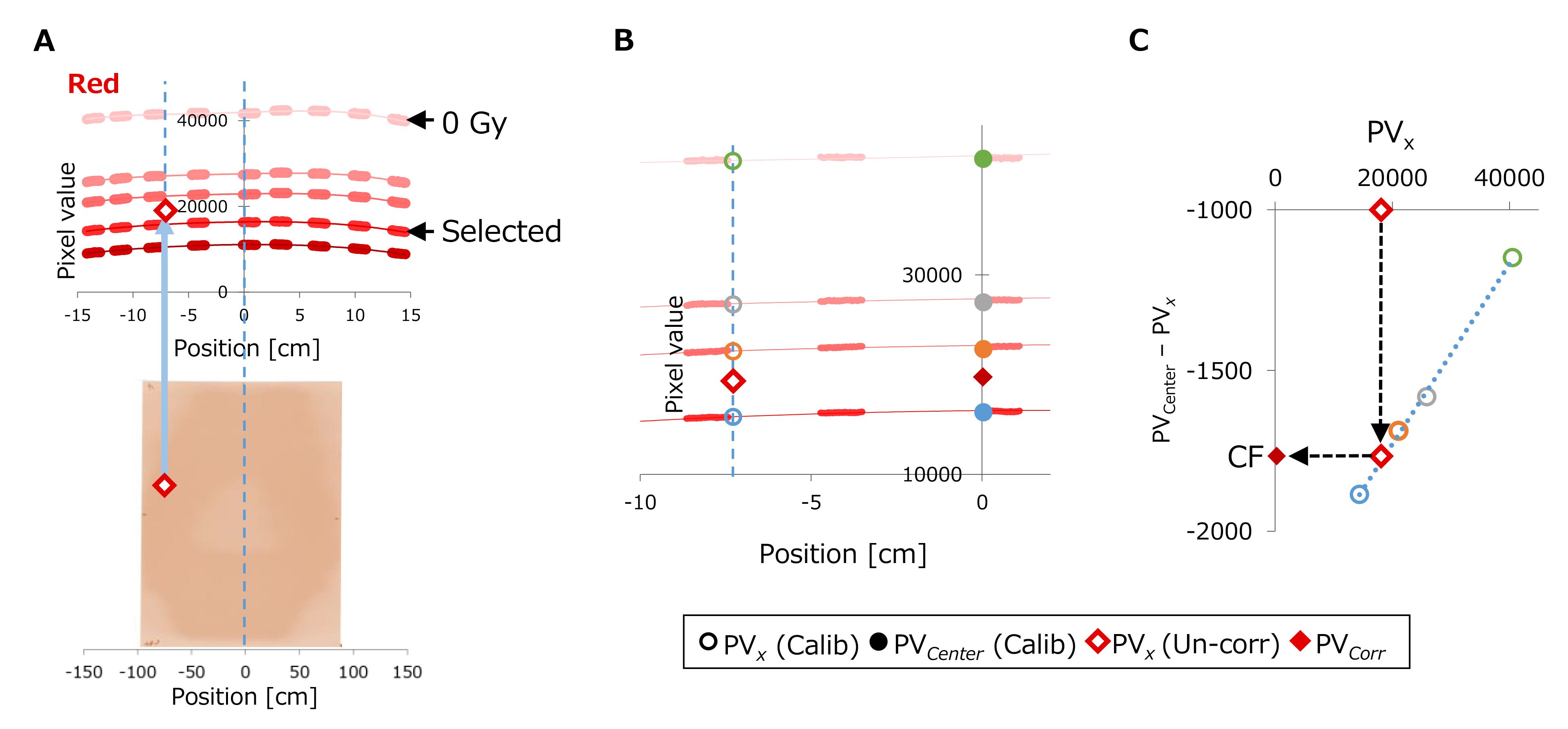

Supplement: Suppl_Figure_3_R1_rraa124 [file suppl_figure_3_r1_rraa124.png]

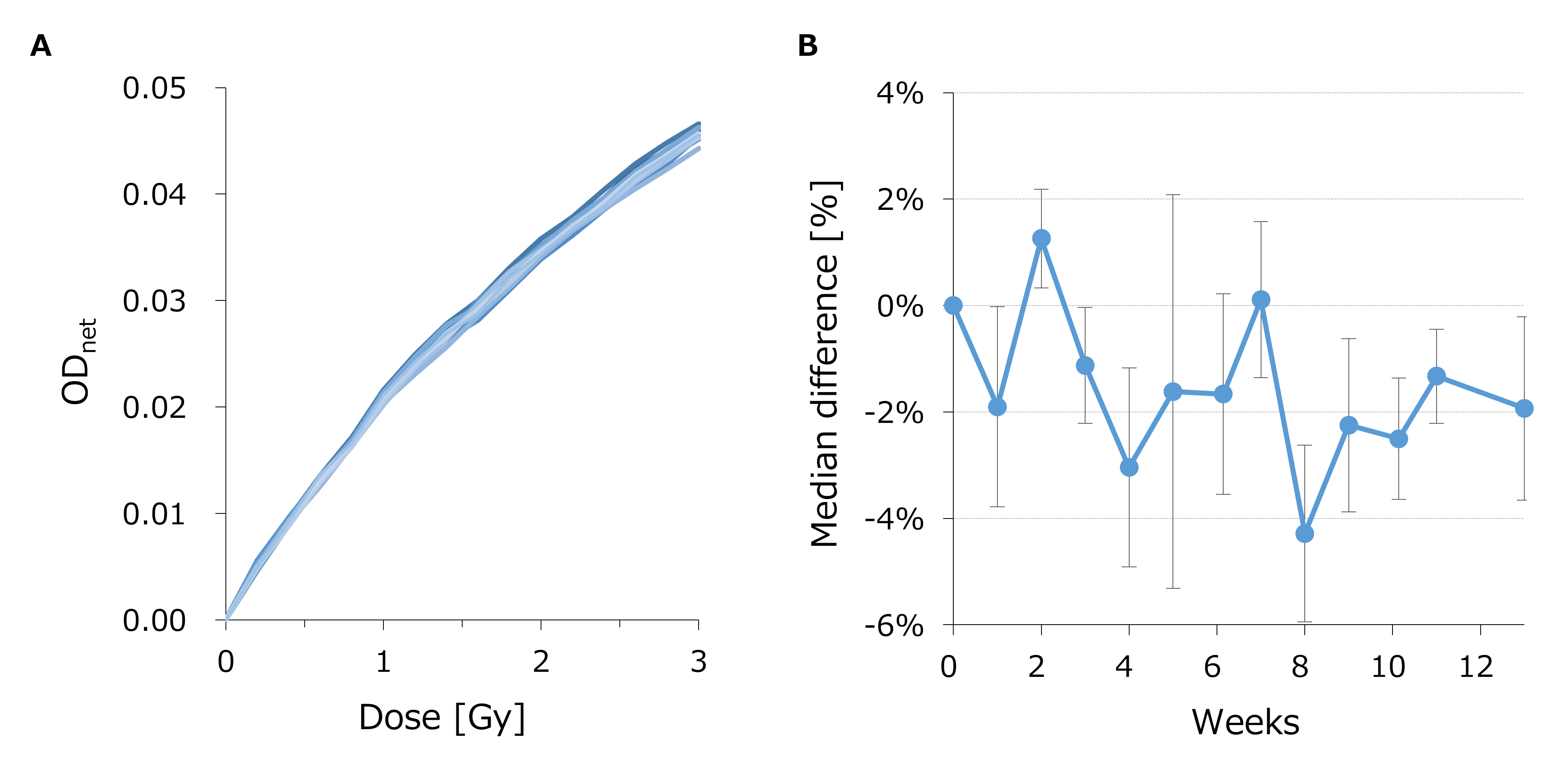

Supplement: Suppl_Figure_4_R1_rraa124 [file suppl_figure_4_r1_rraa124.png]
